# Supplementary material for: A Humanized Yeast Phenomic Model of Deoxycytidine Kinase to Predict Genetic Buffering of Nucleoside Analog Cytotoxicity
Source: Genes (Basel). 2019 Sep 30;10(10):770. doi: 10.3390/genes10100770 (PMC6826991; doi:10.3390/genes10100770)
Supplement: Supplementary file 1 [file genes-10-00770-s001.zip › Additional_Files/Additional_File7_GOTermHeatmaps.zip!/Additional_File7_GOTermHeatmaps/phosphoprotein_phosphatase_activity.pdf]

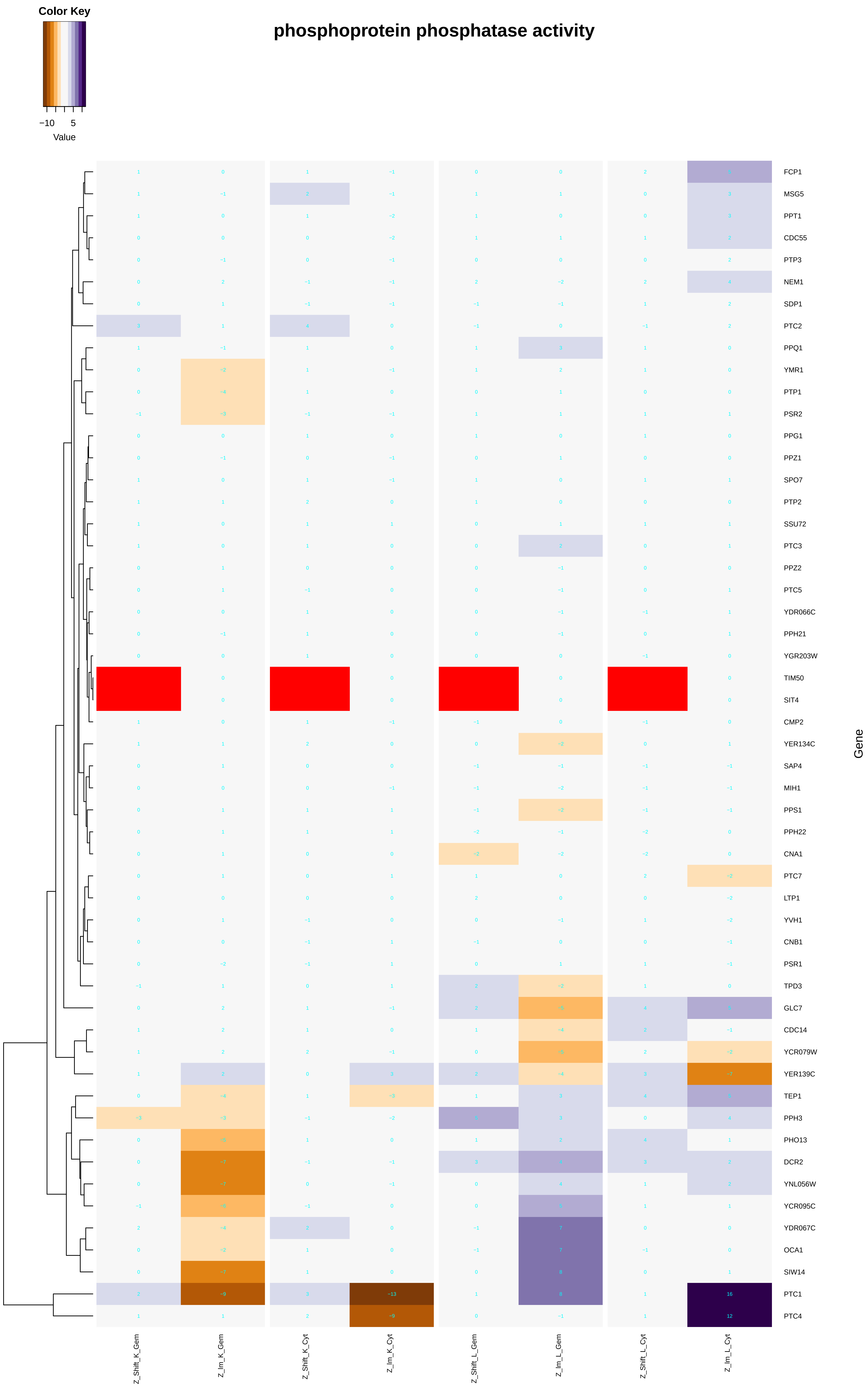
Color Key
−10
5
Value
phosphoprotein phosphatase activity
FCP1
MSG5
PPT1
CDC55
PTP3
NEM1
SDP1
PTC2
PPQ1
YMR1
PTP1
PSR2
PPG1
PPZ1
SPO7
PTP2
SSU72
PTC3
PPZ2
PTC5
YDR066C
PPH21
YGR203W
TIM50
SIT4
CMP2
YER134C
SAP4
MIH1
PPS1
PPH22
CNA1
PTC7
LTP1
YVH1
CNB1
PSR1
TPD3
GLC7
CDC14
YCR079W
YER139C
TEP1
PPH3
PHO13
DCR2
YNL056W
YCR095C
YDR067C
OCA1
SIW14
PTC1
PTC4
Gene
Z_Shift_K_Gem
Z_Im_K_Gem
Z_Shift_K_Cyt
Z_Im_K_Cyt
Z_Shift_L_Gem
Z_Im_L_Gem
Z_Shift_L_Cyt
Z_Im_L_Cyt

protein serine/threonine phosphatase activity

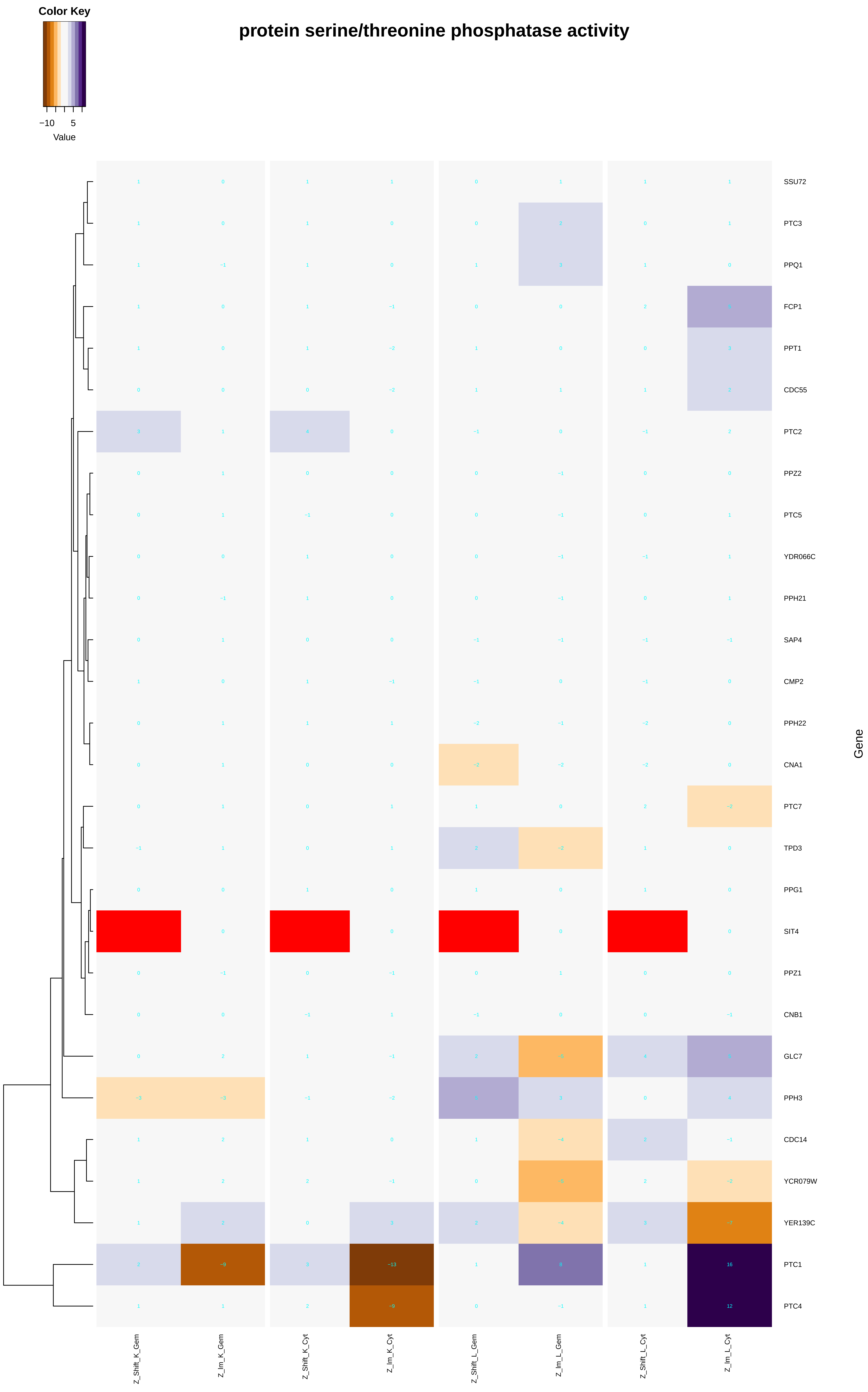

protein tyrosine phosphatase activity

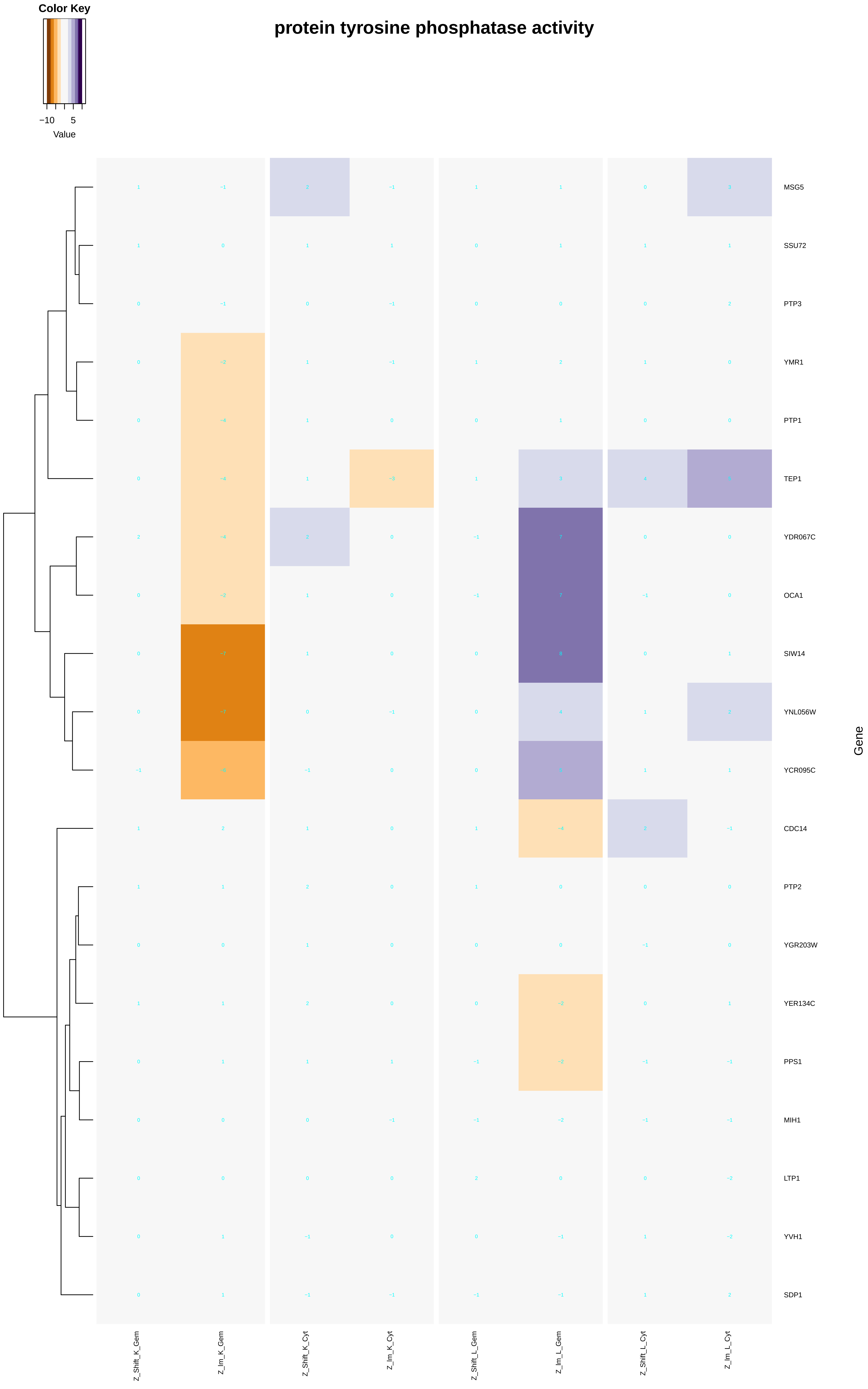

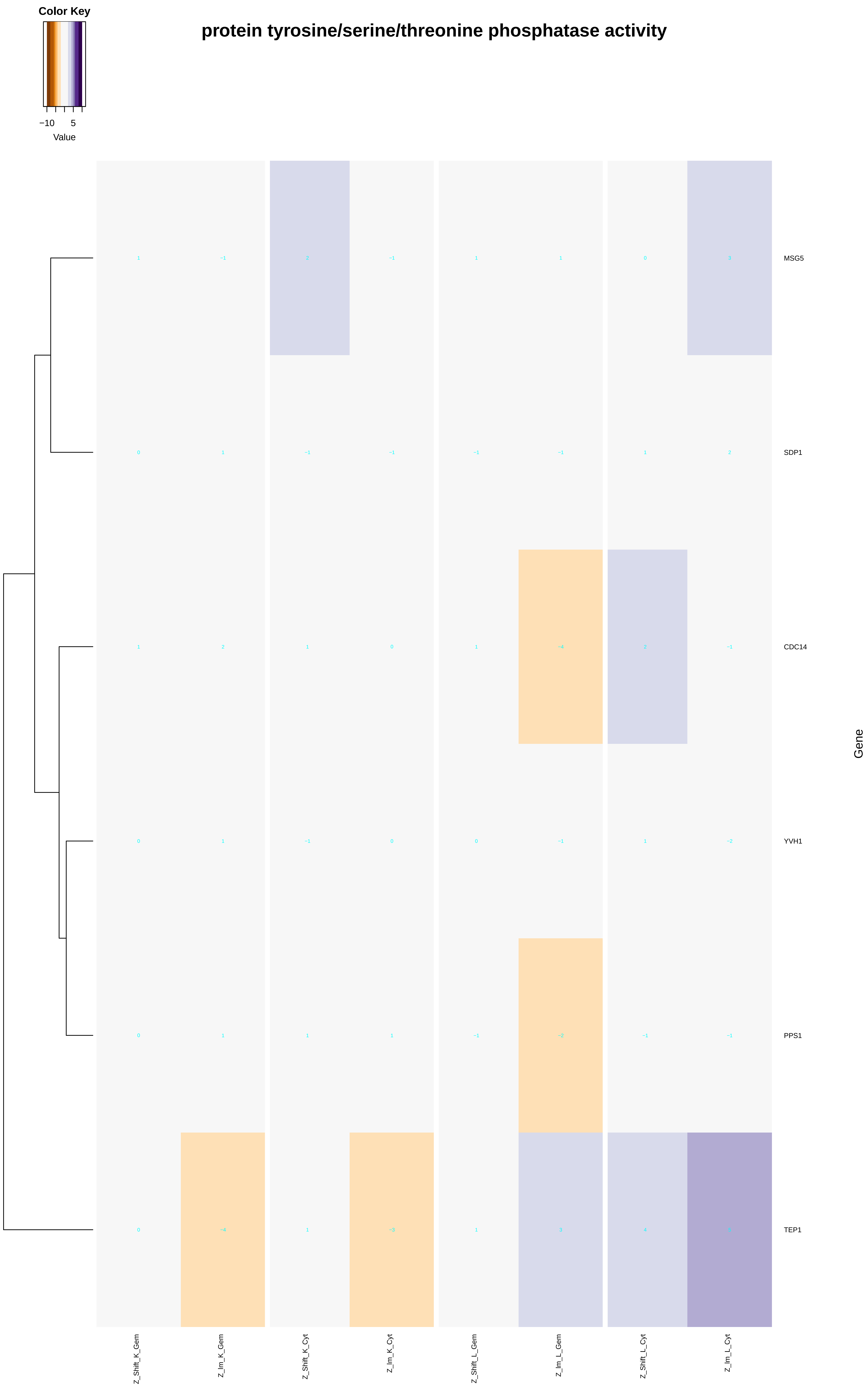
protein tyrosine/serine/threonine phosphatase activity
Color Key
−10
5
Value
MSG5
SDP1
CDC14
YVH1
PPS1
TEP1
Gene
Z_Shift_K_Gem
Z_Im_K_Gem
Z_Shift_K_Cyt
Z_Im_K_Cyt
Z_Shift_L_Gem
Z_Im_L_Gem
Z_Shift_L_Cyt
Z_Im_L_Cyt

calcium−dependent protein serine/threonine phosphatase activity

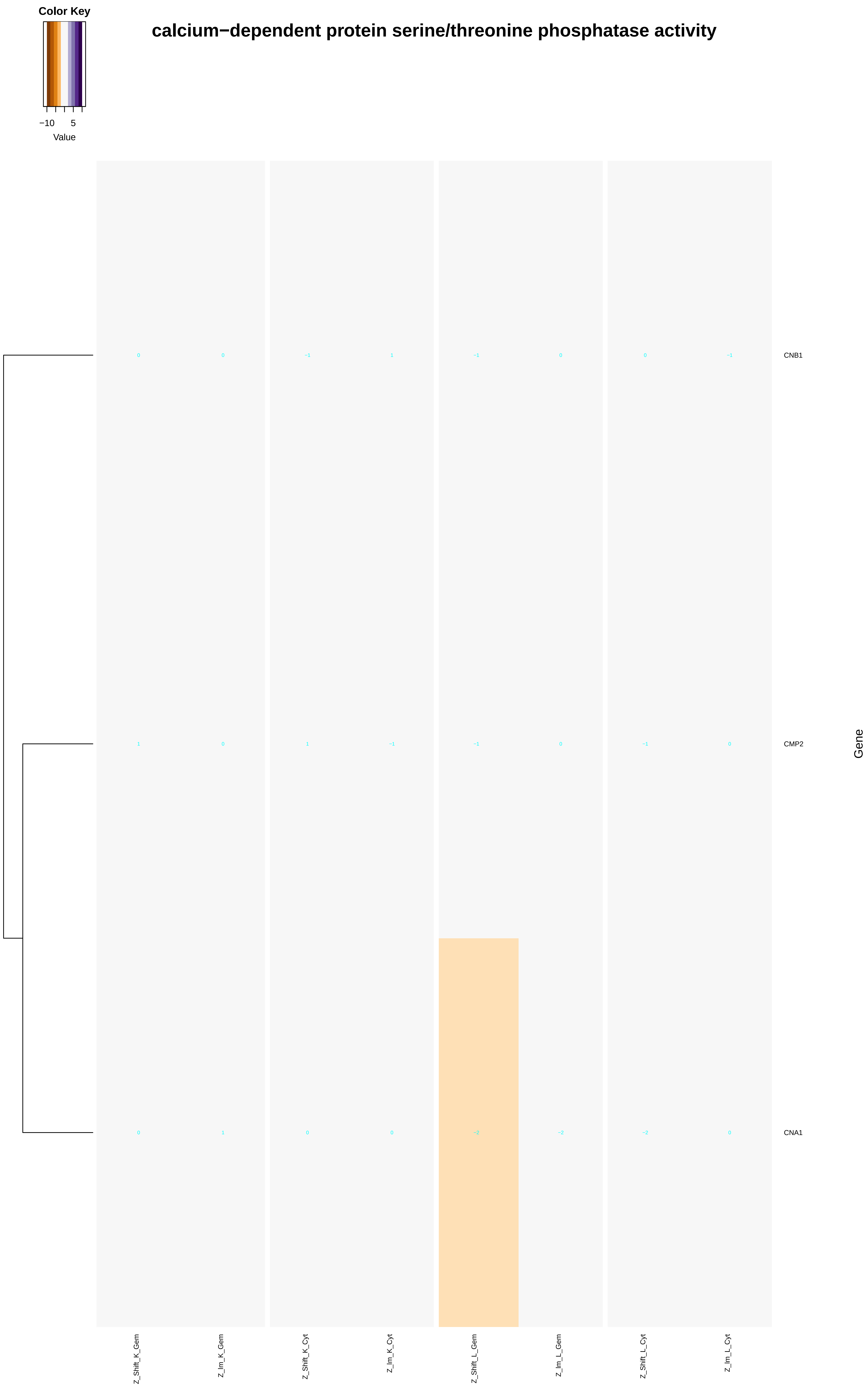

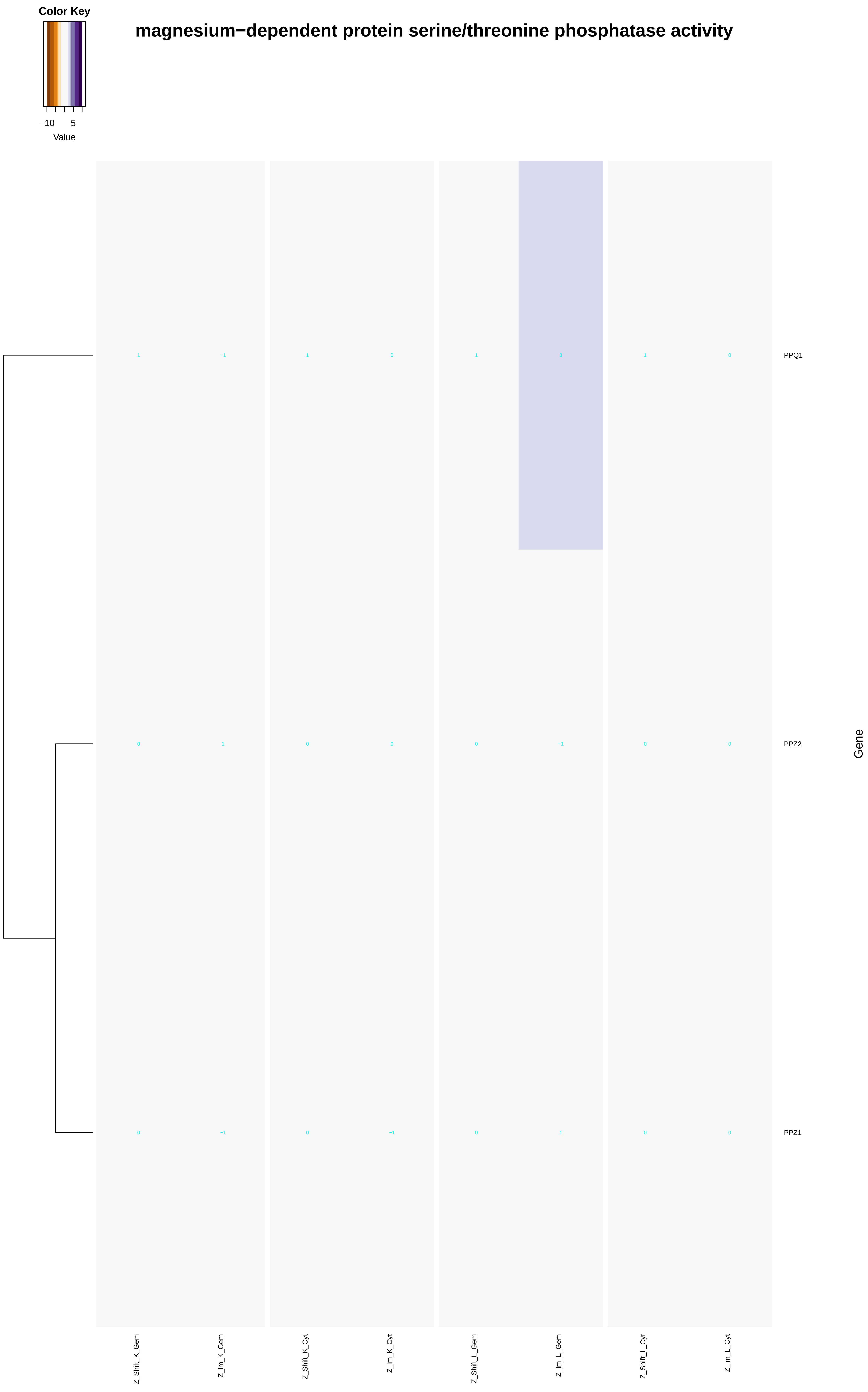
magnesium−dependent protein serine/threonine phosphatase activity
Color Key
−10
5
Value
PPQ1
PPZ2
PPZ1
Gene
1
−1
1
0
1
3
1
0
0
1
0
0
0
−1
0
0
0
−1
0
−1
0
1
0
0
Z_Shift_K_Gem
Z_Im_K_Gem
Z_Shift_K_Cyt
Z_Im_K_Cyt
Z_Shift_L_Gem
Z_Im_L_Gem
Z_Shift_L_Cyt
Z_Im_L_Cyt

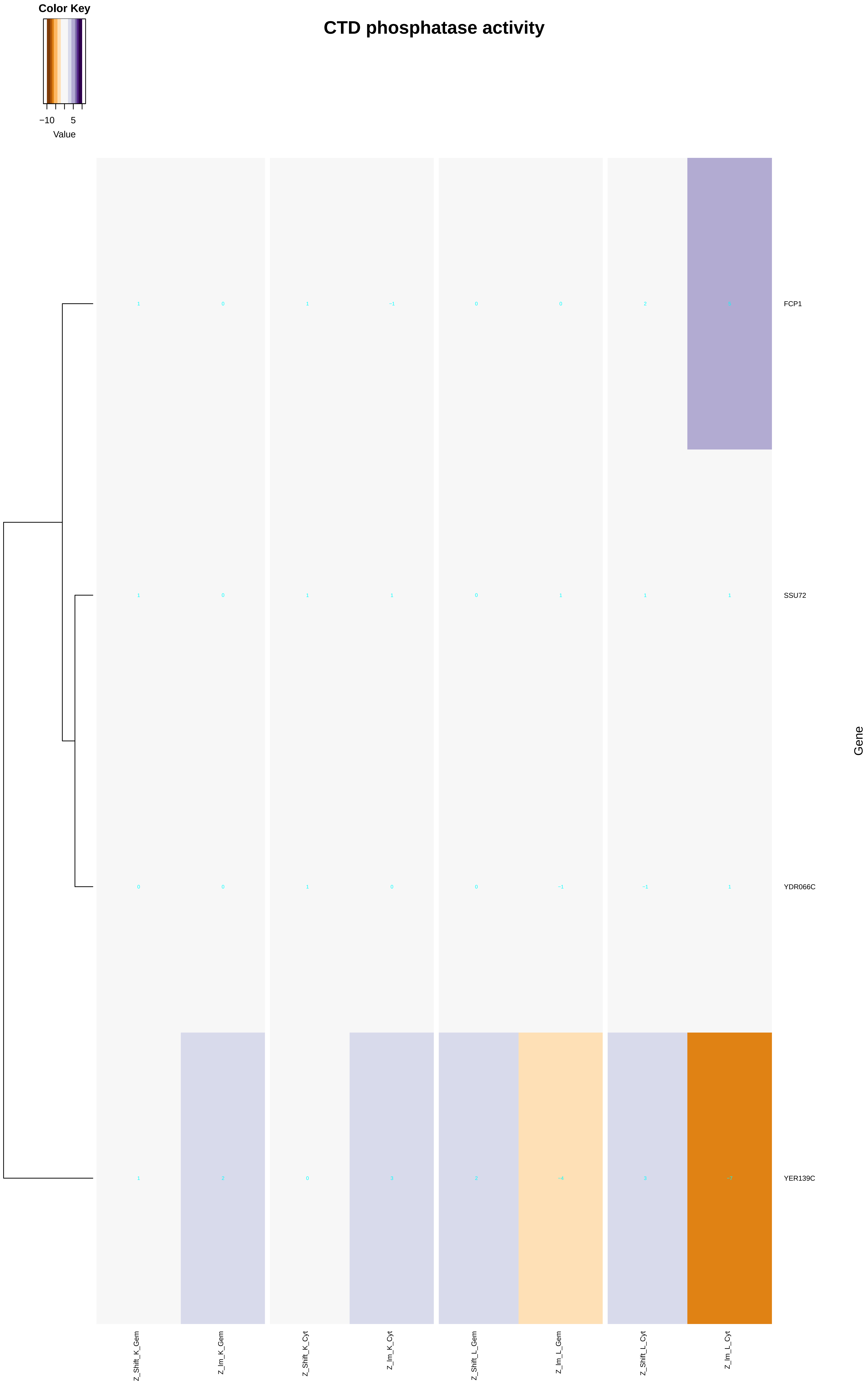
Color Key
−10
5
Value
CTD phosphatase activity
1
0
1
−1
0
0
2
5
FCP1
1
0
1
1
0
1
1
1
SSU72
0
0
1
0
0
−1
−1
1
YDR066C
1
2
0
3
2
−4
3
−7
YER139C
Z_Shift_K_Gem
Z_Im_K_Gem
Z_Shift_K_Cyt
Z_Im_K_Cyt
Z_Shift_L_Gem
Z_Im_L_Gem
Z_Shift_L_Cyt
Z_Im_L_Cyt
Gene
